# Oromandibular Reconstruction: The History, Operative Options and Strategies, and Our Experience

**DOI:** 10.5402/2011/824251

**Published:** 2011-12-12

**Authors:** Pao-Yuan Lin, Kevin C. Lin, Seng-Feng Jeng

**Affiliations:** ^1^Section of Plastic and Reconstructive Surgery, Department of Surgery, Kaohsiung Chang Gung Memorial Hospital, College of Medicine, Chang Gung University, Kaohsiung 83301, Taiwan; ^2^Department of Preventive and Restorative Sciences, School of Dentistry, University of California, San Francisco, CA 94143, USA; ^3^Department of Plastic Surgery, E-Da Hospital, I-Shou University, Kaohsiung 82445, Taiwan

## Abstract

Oromandibular reconstruction resulting from resection of benign tumor, malignant cancer, osteomyelitic or osteoradionecrotic mandible remains a challenge for plastic surgeons today. At present, fibula osteocutaneous flap is the perhaps most commonly used technique for oromandibular reconstruction because of its potential for contouring, immediate dental implant placement, and favorable donor site morbidity. In this study, we review the history of oromandibular reconstruction, summarize the characteristics of different osteocutaneous flaps, offer surgical options of different osteocutaneous flaps, and provide reconstructive strategies for different locations of mandibular defects. Furthermore, we give a detailed description of various modifications in oromandibular reconstruction: (1) the myoosseous flap for lateral segmental defect repair may reduce donor site complication; (2) to improve the function of oral commissure in patients with obscure recipient vessels, we modify the fibula osteocutaneous flap with anterolateral thigh flap and combine the tensor fascia lata using one set of recipient vessel for composite oromandibular reconstruction; (3) to decrease the likelihood of neck infection and improve aesthetic result, we add the segmental soleus muscle to the fibula osteocutaneous flap to obliterate and augment submandibular dead space. Lastly, dental rehabilitation considerations associated with mandibular reconstruction have been given to help assist in surgical treatment planning.

## 1. History of Oromandibular Reconstruction with the Free Osteocutaneous Flap

Taylor and Watson presented the first free groin-iliac crest osteocutaneous flap based on superficial circumflex iliac artery in 1978 [1]. In 1979, he improved the free transfer of iliac crest osteocutaneous flap by involving the deep circumflex iliac artery [2]. In 1994, Koshima et al. [3] further modified the iliac crest osteocutaneous flap by using the lateral circumflex femoral artery to repair large mandibular defects.

 Taylor et al. [4] first introduced the fibula flap in 1975, but it did not become popular for mandibular reconstruction until after 1989 when Hidalgo [5] utilized this technique to restore 12 mandibular defects. Since then, multiple modifications and applications of the fibula flap have been proposed. Zlotolow and colleagues incorporated secondary osseointegrated dental implants for functional rehabilitation in 1992 [6]. Wei and colleagues described the use of osteoseptocutaneous fibula flap for reconstructing composite mandibular defects in 1994 [7]. In the same year, Wei et al. and O'Leary et al. independently sought to convert the fibula flap into a neurosensory flap by incorporating the lateral sural cutaneous nerve [8, 9]. Most recently, in 2010, Kuo and colleagues combined partial soleus muscle with fibula osteoseptocutaneous flap for dead space obliteration [10].

 While the fibula osteoseptocutaneous flap has probably become the workhorse flap in mandibular defect reconstruction, various alternative techniques deserve much attention from the surgeons as well. The radial forearm flap, also known as the Chinese flap, was first introduced by Yang et al. [11]. In 1983, Soutar et al. [12] popularized the technique in his clinical study and advocated for its use in mandibular reconstruction. In 1986, Swartz and Banis Newton [13] proposed a different technique to restore mandibular and maxillary defects using the scapula osteocutaneous flap. In 1987, without using any bone flap to maintain the continuity of the mandible, Klotch and Prein [14] reported using AO reconstruction plate to repair mandibular defects along with an additional free flap for skin or soft tissue coverage. Finally, in 2007, Kim and Blackwell [15] proposed the use of the latissimus-serratus-rib flap for oromandibular and maxillary reconstruction.

## 2. Indications of Oromandibular Reconstruction

Complex oromandibular tissue defects can often be corrected with a single operation following tumor resection. Primary reconstruction of mandibular defects is usually performed immediately at the time of benign tumor ablation (e.g., ameloblastoma), malignant cancer extirpation, or the resection of osteomyelitic and stage III osteoradionecrotic mandible. Primary repair of oromandibular defects offers significant advantage over secondary repair by preventing the wound from scarring while obtaining optimal functional and aesthetic results for the patient. Secondary reconstruction of mandibular defects is usually not recommended unless previously reconstructed tissue develops persistent infection and postoperative complications (i.e., screw loosening or plate extrusion). Secondary reconstruction presents a unique challenge for the surgeons due to the presence of soft tissue scarring and the contracture of the resected end of the mandibular tissue. This often hinders the surgeon's ability to predict the length and the amount of mucosa required intraorally.

## 3. The Goals of Oromandibular Reconstruction

The primary goals of oromandibular reconstruction are to achieve primary wound closure as well as to obtain a functional and aesthetic restoration [16]. First, to avoid infection and facilitate wound healing, the use of soft tissue coverage (regional pedicled or free skin flap) helps establish primary wound closure in oromandibular reconstruction. Secondly, to obtain a functional and aesthetic restoration, it is important to reconstruct the intraoral lining using a reliable and practical flap to drape over the alveolus and the floor of the mouth. The thin flap facilitates wound healing, allows tongue movement, and avoids flap redundancy. Finally, when utilized in conjunction with dental implants, free vascularized bone flap using the fibula, the iliac crest, the scapula, and the radius may be effective in restoring the mandible both functionally and aesthetically. One must note that reestablishing the hard tissue continuity is critical for the ultimate success of the reconstruction.

## 4. Reconstructive Options

With the advancement in microsurgery, free flap transfer can effectively diminish the challenges of restoring extensive oromandibular defects subsequent to tumor resection, cancer recurrence, and radiotherapy complications. Free anterolateral thigh (ALT) flap is the most commonly used technique in our institution for intraoral lining repair because of its reliable vascularity, easy harvest, and minimal donor site morbidity. However, one must remember that the ALT flap is merely a soft tissue repair; without maintaining the mandibular continuity, the functional and aesthetic outcome of the reconstruction may remain compromised. Combining reconstruction plate with regional pedicled or free skin flap may be a viable alternative for maintaining mandibular continuity; but, given the limitations, it is currently only accepted for the reconstruction of lateral mandibular defects [14].

The free vascularized iliac crest was first described for the reconstruction of mandibular defects in 1979. Since then, the free osteocutaneous flap has become the gold standard reconstructive technique for the repair of oromandibular defects. Today, the most commonly used free flaps for mandibular reconstruction are the fibula, the scapula, the iliac crest, and the radial forearm osteocutaneous flap. Each of these free flaps will be described in more detail in the following sections. The summarized characteristics of these four flaps are listed in Table 1.

### 4.1. The Fibula Flap

Since Hidalgo proposed the use of the fibula flap for the reconstruction of mandibular defects in 1989 and Wei et al. [7] popularized the use of the fibula osteoseptocutaneous flap in 1994, the free fibula osteocutaneous flap has become the flap most commonly used in oromandibular reconstruction. The peroneal artery provides circulation to the skin on the lateral aspect of the leg through the musculocutaneous and septocutaneous branches. In a cadaveric injection study, 1 or 2 sizable perforators were discovered in the posterior crural septum that could supply the vasculature of the skin paddle approximately 22 to 25 cm in length and 10 to 14 cm in width [7]. Clinically, the peroneal artery can sufficiently supply the endosteal and periosteal circulation of the fibula, thus making multiple osteotomies of the flap possible. This allows the surgeon to contour the bone according to the shape of the defect and reach maximum aesthetic outcome. In terms of circulatory consideration, if there is a history of prior trauma or arteriosclerosis, a lower extremity arteriogram would be required to assess if the patient is suitable for such a flap procedure.

**Figure 1:**
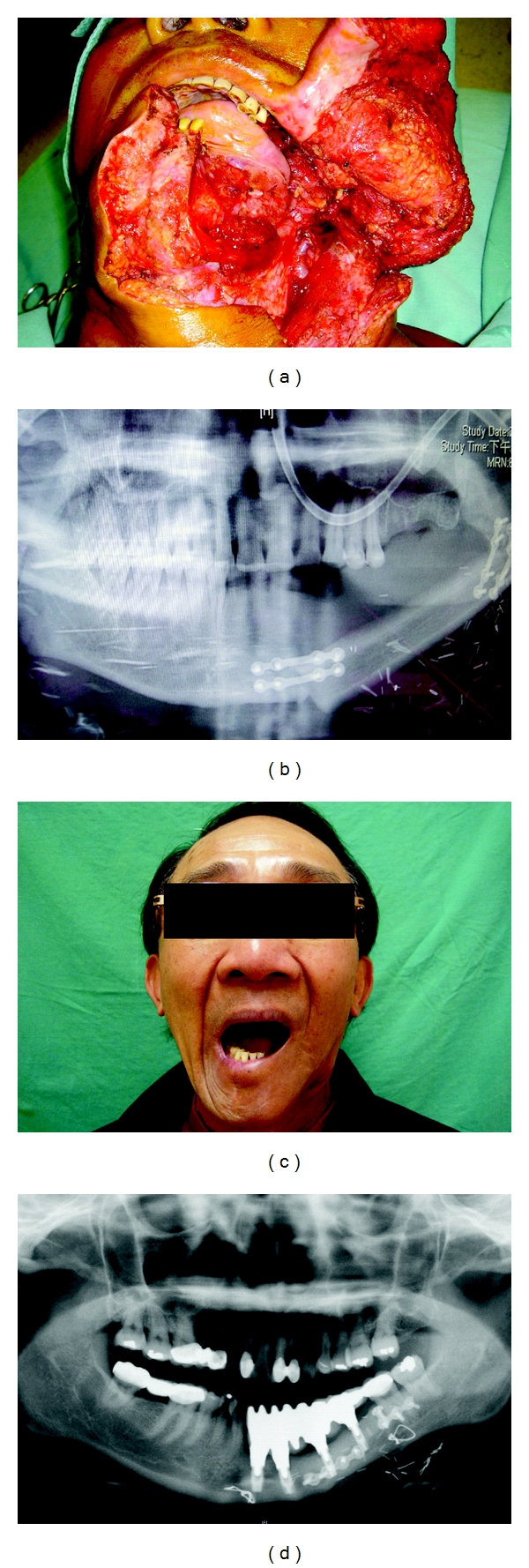
(a) The patient with anterolateral oromandibular defect following lower gum cancer ablation. (b) The defect was reconstructed by fibular osteocutaneous flap. Miniplates were used for bony fixation. (c) Good aesthetic result and adequate moth open were shown in this patient after fibula flap reconstruction. (d) Osseointegration of dental implant was suitable followed by fibula flap reconstruction.

 Another advantage of the fibula flap lies within its bone architecture. Similar to the mandible, the fibula possesses a bicortical layer and provides similar bone support for dental implants. This makes the fibula a suitable mandible substitute functionally. If coverage of the floor of the mouth is needed, a thin skin paddle can also provide an adequate aesthetic result (Figures 1(a)–1(d)). Since the fibula is distant from the oral cavity, the reconstructive team and the extirpative team can simultaneously perform the flap harvest and tumor resection at the same time and significantly decrease the operating time.

Finally, with regard to the donor site morbidity, nearly all patients are able to engage in daily and recreational activities besides having minor complaints such as ankle stiffness or numbness of the lower leg [17].

### 4.2. The Scapula Flap

The vascular supply of the scapula flap is based on the circumflex scapular artery, a branch of the scapular artery. The circumflex scapular artery gives off several branches to supply the periosteum and muscles such as the long head of the triceps, the teres major, and the teres minor. The scapular vascular supply consists of a rich fascial layer with vertical perforators as well as a subcutaneous vascular plexus.

 The lateral border of the scapula provides a straight segment of corticocancellous bone measuring 1.5 to 3 cm in thickness and 10 to 14 cm in length. When the tip of the scapula is included, an additional 3 to 4 cm along the medial border of bone is also available for the reconstruction of the mandibular angle without an osteotomy [13].

The advantages of the scapula flap are the superior color match of the skin paddle and the possibility of harvesting large multiple skin paddles from the posterior thorax. In contrast, the drawback of the scapula flap is that the patient needs to be repositioned during the surgery.

### 4.3. The Iliac Crest Flap

The vascular supply of the free iliac crest osteocutaneous flap is based on the deep circumflex iliac artery (DCIA) that supplies the soft tissue and the bony wall of the iliac fossa. More specifically, the DCIA supplies the bone through many tiny foramina on the medial aspect of the crest and the blade of the ilium.

In terms of soft tissue vascular supply, previous cadaveric injection studies indicated that the musculocutaneous branches of the DCIA could supply an area of skin flap ranging from 10 × 7 cm to 30 × 15 cm. However, clinically, the skin paddle of this flap usually appears too bulky and unsuitable for the reconstruction of inner lining defects. In addition, for external coverage or through-and-through defects, the color match of this flap may also appear inferior compared to other flaps.

 The advantage of iliac crest bone flap is that a large block of bone graft can be harvested. The shape of the iliac crest makes it suitable for reconstructing the mandible especially at the anterior curvature or the symphysis. And because of the large block of graft available, the height is also adequate for the placement of dental implants. Furthermore, if sensory reinnervation of the flap is desired, the lateral branch of the last thoracic nerve may be used because of its sufficient size. While the iliac crest flap offers much advantage, the disadvantage is its significant donor site morbidity such as severe postoperative pain, contour irregularity, and iatrogenic hernia.

### 4.4. The Radial Forearm Osteocutaneous Flap

Before selecting the radial forearm osteocutaneous flap for grafting, the Allen's test of the donor hand should be performed preoperatively to confirm the presence of adequate circulation to the hand via the ulnar artery. The quality of the skin paddle of the radial forearm osteocutaneous flap is superior to the fibula flap because of its thinness and flexibility. The length of the pedicle is also long enough to provide microanastomosis without any vein grafts. If sensory reinnervation is desired, the anterior antebrachial cutaneous nerve may be incorporated in the flap. In terms of the disadvantage, the bone quality is inferior to the fibula.

In oromandibular reconstruction, this particular flap is indicated only when a relatively small volume of bone is required. The radial forearm flap is helpful for the localized reconstruction of the ascending ramus, the angle, and the posterior non-tooth-bearing regions of the mandible especially when a soft tissue lining is required. This flap is inadequate for repairing large volume or contour defects; the lack of bicortical fixation also makes the flap unsuitable for the placement of dental implants. Lastly, the potential donor site morbidity of the radial forearm osteocutaneous flap is radial fracture.

## 5. Reconstructive Strategies

**Figure 2:**
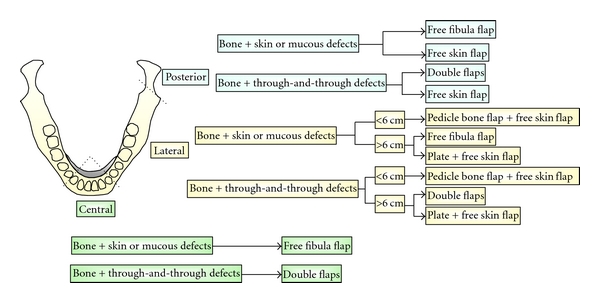
Classification of oromandibular defect and recommended surgical strategies in different regions of oromandibular defect.

In order to achieve a normal contour for the mandible, multiple osteotomies are often required. Subsequently, the surgical strategies for reconstructing the mandibular bony defects often vary according to the location and the size of the defects. For ease of differentiation, we modify the classification of mandible defects into central defects (C), true lateral defects (L), and posterior defects (P). The approach to reconstructing the mandibular bony defects is based on the modified CLP classification. The recommended surgical strategies of oromandibular reconstruction in different locations are depicted in Figure 2.

The border of central segmental defects (C) is defined by the involvement of the mandibular midline, the four incisors, and the two canines. For central (anterior) segmental defects and in younger patients where dental implant therapy is involved, the free orocutaneous flap is the gold standard technique. In larger defects, reconstruction plate is used for bone fixation following osteotomies; in smaller defects, miniplates are used because they limit the elevation of the periosteum and minimize the risk of compromising the bone vasculature. The use of reconstruction plate alone is not recommended for restoring anterior segmental defects. Schusterman et al. [18] reported high failure rate (4 of the 20 studied patients) in restoring anterior segmental defect reconstruction with reconstruction plate. In Boyd et al.'s study [19], although the overall success rate of mandibular plate reconstruction was 78.9%, high failure rate (35%) was also exhibited in anterior defects.

 The border of lateral segmental defects (L) is defined by the body and/or the ramus of the mandible without involving any portion of the central segment. With regard to the reconstruction, the surgical modalities include the free osteocutaneous flap, reconstruction plate combined with soft tissue transfer, and pedicled vascularized myoosseous flap combined with free skin flap transfer. When comparing the surgical outcomes of using the free vascularize flap versus the use of reconstruction plate, Shpitzer et al. [20] concluded that, for lateral mandibular defects, the osteocutaneous free flap presented with better long-term prognosis than that of the reconstruction plate. Furthermore, additional studies have indicated that, on average, 5% of patients may suffer from fractured reconstruction plate [21]. Subsequent to similar clinical findings, for patients possessing relatively good life expectancy who are concurrently receiving radiotherapy, the use of reconstruction plate combined with free skin flap transfer (e.g., free radial forearm flap or ALT flap) has become less acceptable clinically.

 The border of the posterior defects (P) is defined by the involvement of the condyle and the ramus up to the angle of the mandible. The reconstructive option is to either utilize the free osteocutaneous flap or the free tissue transfer alone. Hanasono et al. [22] compared the outcomes of these two modalities in restoring posterior mandibular defects they found that, while soft tissue free flap alone may achieve reasonable aesthetics, masticatory function, and degree of mouth opening, in young patients with good surgical prognoses, the vascularized bone flap is preferred because of the more predictable dental function.

## 6. Prognosis of the Free Osteocutaneous Flap Reconstruction

For oromandibular reconstruction, particularly those with anterior or large bony defects requiring multiple osteotomies, many articles have demonstrated high success rate and good functional and aesthetic results using various osteocutaneous flaps [20, 23–28]. The most commonly used parameters for outcome assessments are restoration of diet, intelligibility of speech, and aesthetics. The outcome assessments of osteocutaneous flap reconstruction are summarized in Table 2.

## 7. Donor Site Complications following Free Fibula Harvest 

As reported by Anthony et al. [17], 17% of donor site presents with immediate complications such as wound infection and skin graft loss. While such incidence may be considered relatively low, we should not underestimate the significance of late morbidities such as pain, edema, ankle stiffness, ankle instability resulting from peroneus longus muscle weakness [29] and foot numbness [30]. Despite the effort to preserve ankle function by reconstructing the donor site using split fibular bone, via quantitative analysis, Hsieh and colleagues found that the range of motion in patients' plantarflexion and dorsiflexion and the strength of their knees and ankles remained significantly compromised [31]. Furthermore, Babovic et al. [32] reported that although the majority of patients were able to engage in daily routine after rehabilitation, these patients may encounter limited ambulation distance in the short term.

## 8. Authors' Experiences in Oromandibular Reconstruction 

### 8.1. Pedicled Myoosseous Flap in Conjunction with Free Skin Flap Transfer for Lateral Segmental Defect Reconstruction [33]

#### 8.1.1. Indication

**Figure 3:**
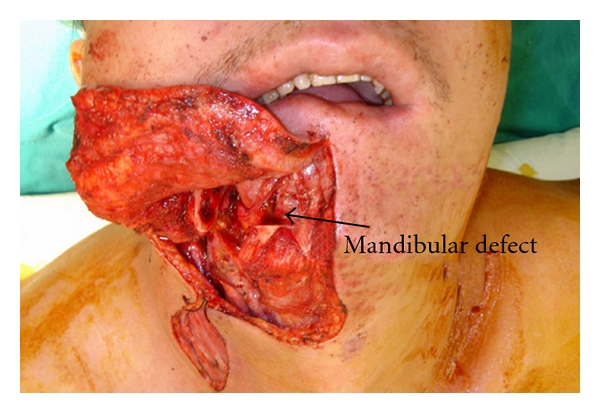
The patient had lateral mandibular defect that was less than 6 cm in length.

When the lateral segmental mandibular defect is less than 6 cm (Figure 3) or when the patient does not wish to sacrifice his/her fibula bone, pedicled myoosseous flap is an alternative option for obtaining the continuity of mandible.

#### 8.1.2. Surgical Anatomy

**Figure 4:**
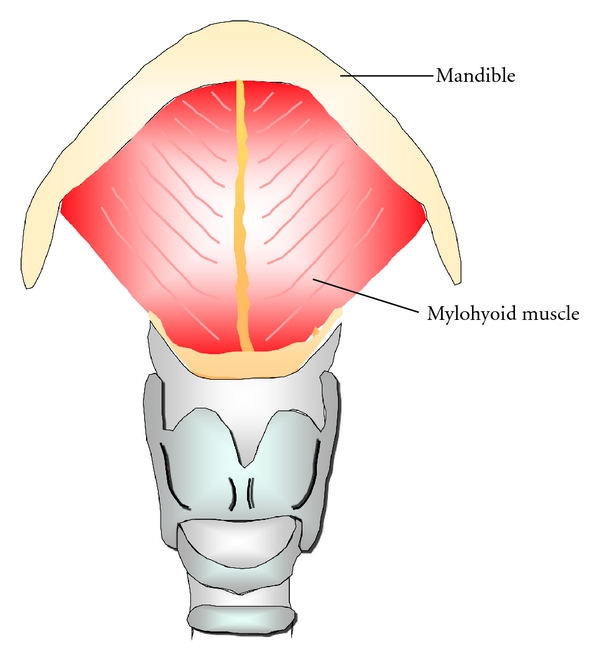
Mylohyoid muscle provides the blood supply of lower medial aspect of the mandible.

**Figure 5:**
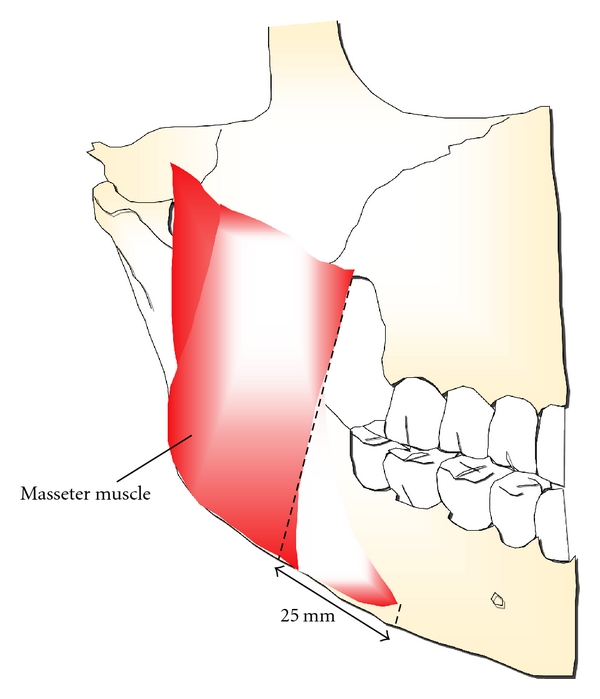
The lower anterior border of the deep masseter provides the blood supply of lateroposteral aspect of the mandible.

The blood supply for pedicled myoosseous flap is based on the mylohyoid muscle anteriorly and the masseter muscle posteriorly. The mylohyoid muscle is thin and flat. It originates from the mylohyoid ridge on the medial aspect of the mandible and inserts into the body of the hyoid bone (Figure 4). The masseter muscle is thick, quadrilateral in shape, and consists of superficial and deep parts. The lower anterior border of the deep masseter is curved as the muscle extends about 25 mm from the projection of the superficial masseter (Figure 5) and provides the blood supply of lateroposteral aspect of the mandible.

#### 8.1.3. Horizontal Osteotomy Procedure

**Figure 6:**
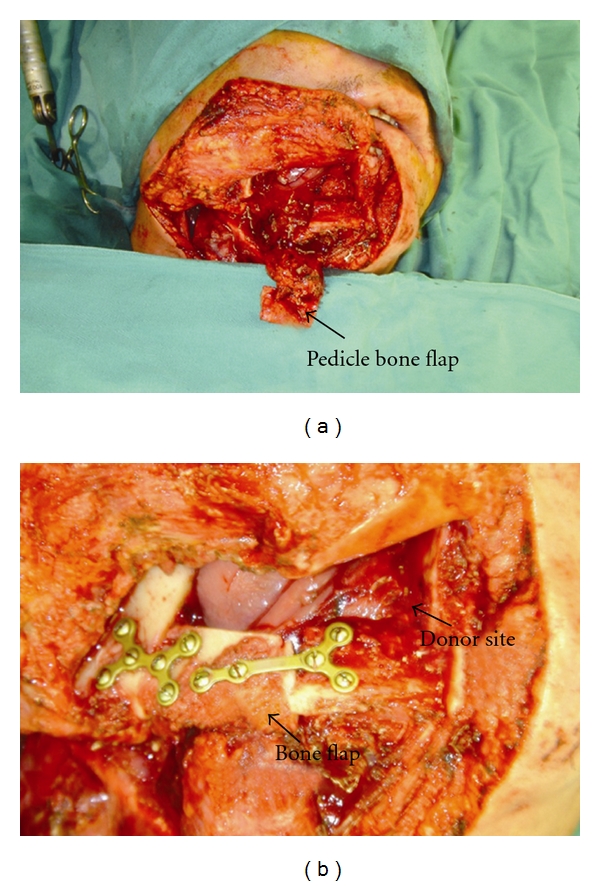
(a) The pedicle bone flap that was supplied by mylohyoid muscle was harvested. (b) The bone flap was transferred posteriorly and attached to the posterior mandibular bone with miniplates.

Osteotomy is carried out on the remaining mandibular body. The bone cut is made parallel to the lower mandibular margin and should be 7.5 mm above the inferior border of the mandible in order to avoid damaging the inferior alveolar nerve. Also, to avoid damaging roots of the teeth, the cut should preserve at least 2 cm segment of the alveolar bone (Figures 6(a) and 6(b)).

Finally, the attachment of mylohyoid muscle on the medial aspect of mandible at the time of osteotomy should be preserved. For larger bone defects, the second osteotomy may be necessary in the remaining mandibular angle area. In this instance, the insertion of the lower anterior border of masseter should be carefully preserved to maintain vascularity. This masseter-based bone flap can be advanced anteromedially to attach to the mylohyoid-based bone flap and allow reconstruction of the complete bone defect.

#### 8.1.4. Clinical Outcomes

12 patients with lateral segmental mandibular defects received the pedicled myoosseous flap (8 single, 3 double, 1 sagittal osteotomy) combined with free skin flap reconstruction (9 ALT flaps and 3 radial forearm flaps). All of the patients' newly reconstructed mandibles have acceptable contour. The bone scan performed one week postoperatively confirmed viable bone flaps in 11 cases. 10 patients were able to restart soft to full diet. In this series of cases, a patient experienced failed ALT flap and 2 patients' bone flaps were nonviable.

### 8.2. Reconstruction of Extensive Composite Mandibular Defects with Large Lip Involvement Using Double Free Flaps and the Fascia Lata Grafts for the Oral Sphincters [34]

Extensive composite mandibular defects involving large lip defects remain a significant challenge among head and neck reconstruction today. The senior author (SJ) used the fibula osteocutaneous flap for oromandibular repair, the ALT flap for external cheek defect reconstruction, and the tensor fascia lata for the substitute of the oral sphincter in 10 studied patients. For patients without 2 sets of obvious recipient vessels, flow-through microvascular anastomosis was performed (i.e., the pedicle of the second free flap (ALT flap) can be attached to the distal runoff of the first fibula flap) [35]. The flaps had 100% survival rate. All the patients were able to restart on a soft diet. The speech intelligibility was nearly normal for all the patients and all the patients had gained an acceptable appearance.

### 8.3. Free Fibula Osteocutaneous Flap with Soleus Muscle as a Chimeric Flap for Reconstructing Mandibular Segmental Defect after Oral Cancer Ablation [10]

The skin paddle of the fibula osteocutaneous flap is sufficient for covering the intraoral lining or outer cheek defects. However, these flaps appeared inadequate for soft tissue augmentation of the submandibular area and often led to obliteration of dead space following lymph node dissection and tumor ablation. Therefore, the ideal flap for intraoral reconstruction should be pliable and provide sufficient bulky tissue to fill the dead space. We used the fibula osteocutaneous flap with a segment of the soleus muscle to reconstruct the oromandibular defects and obliterate the submandibular dead space in 20 patients with oral cancer. One flap failed due to venous insufficiency. All patients resumed a soft diet, spoke intelligibly, and had a satisfactory facial contour at a mean 12-month followup. No patients had any major donor foot complications and all were able to stand on the balls of the feet without difficulty postoperatively.

## 9. Dental Rehabilitation Considerations 

Subsequent to mandibular reconstruction, dental rehabilitation helps to restore facial appearance, speech, and masticatory function that are critical for nutritional intake and the psychological well-being of our patients [36]. Advancement in microsurgery has allowed surgeons to adequately restore bony and soft tissue defects, jaw continuity, and tongue shape. However, limited by the lack of tissue support, mandibular reconstruction often results in compromised dental rehabilitation [37]. While dental implants may be used to facilitate retention, stability, and support for the prostheses, the selection of their uses must also be cautiously evaluated. In this section, we will discuss the masticatory function associated with mandibular reconstruction as well as the indication and timing for dental implant therapy.

### 9.1. Masticatory Function Evaluation and the Role of Dental Prosthesis

Normal masticatory function involves the manipulation, trituration, and consolidation of a food bolus before deglutition occurs. Following a mandibulectomy, the trituration phase of mastication is often significantly affected by the loss of mandibular structure, altered maxilla-mandibular relationships, and decreased tooth-to-tooth contacts [38]. In a cross-sectional study, Curtis and colleagues evaluated the masticatory function of mandibulectomy patients with reconstruction and without reconstruction. Based on the tongue and cheek function measures, the reconstructed group had significantly higher level of masticatory function compared to the nonreconstructed group [38]. While the occlusal force may be poorly correlated with function, the neuromuscular balance between the tongue and soft tissue in mastication is critical. Loss of tongue bulk and immobility of the residual tongue element may inhibit the patient from articulating, swallowing, and manipulating saliva, food bolus, and dentures. Considering the significance of tongue function in mastication, surgeons may consider using free flaps (radial forearm, fibular, scapular, etc.) to replace missing portion of the anterior tongue because of their improved tissue flexibility [39]. Lastly, to examine the efficacy of prosthodontic intervention in patients with mandibular reconstruction, Roumanas et al. found that both conventional prostheses and implant-supported prostheses could restore masticatory function to presurgical levels. And, compared to conventional prostheses, the implant-supported prostheses contributed to greater support and stability and led to improved mastication and superior performance on the defect side [37].

### 9.2. Implant Therapy Considerations

The use of endosseous implants in free flap mandibular reconstruction has significantly reduced the prosthetic problems relating to the retention and stability of conventional prostheses and in patients with reduced salivary flow [40]. In terms of patient selection, inclusion for implant therapy after mandibular reconstruction may be based on several factors. First, the primary objective of dental rehabilitation is to restore the patient's masticatory function to the presurgical state. The rehabilitation is not meant to improve upon the preexisting dental deficiency prior to reconstructive surgery. Secondly, patients must possess sufficient residual oral functions such as the required distance of mouth opening for instrument access, sufficient tongue function, adequate maxilla-mandibular relationship, and lip competence. Finally, the patients must be motivated to adhere to strict oral hygiene requirement and be prepared for the need of preprosthetic and postprosthetic procedures if necessary [40].

Traditionally, the placement of dental implants is usually performed as a secondary procedure following the reconstruction surgery and a latency period. Implant placement is typically not performed during the reconstruction for several reasons: (1) the position and angulation of the placed implants may be compromised, thus leading to prosthetic challenges later on; (2) an increased reconstruction time may lead to increased anesthesia duration and graft morbidity; (3) the graft itself may experience failure or that in case a malignant tumor, additional resection may be necessary due to recurrence [40].

To address some of the concerns, the senior author and coworkers have developed a treatment protocol that allows for primary placement of dental implants during reconstruction surgery [41]. First, the topography of the implant position is accentuated by connecting the waxing screws onto the fixtures during reconstruction. This allows adjustment of the reconstructed bone segment and avoids inadvertent tilting of the graft-implant construct during fixation. Secondly, by using the two-team approach in which graft harvest begins at the same time as the resection, the entire surgical time is reduced to less than 4 hours. Without compromise, this leaves ample time to perform the osteotomies, place implant fixtures, and complete the vascular anastomosis for flap survival. Lastly, in order to avoid the high potential for tumor recurrence, the extirpative team extends the resection to involve 1 to 2 cm of uninvolved surrounding cancellous bone following recommendations proposed by Feinberg and Steinberg.

## 10. Conclusion 

While the fibula osteocutaneous flap, our choice of flap, allows for osteotomy and placement of dental implants, we present various modifications to improve the oral sphincter, reduce postoperative wound infection, and reduce donor site morbidity. The improvements include pedicled myoosseous flap with free skin flap, double free flaps with the tensor fascia lata for composite oromandibular defect reconstruction, and the fibula flap with a segmental soleus muscle for augmentation of submandibular dead space.

 In this study, we examine the prognosis of various surgical modalities in different oromandibular defect locations. To achieve a functional and aesthetic outcome, the free osteocutaneous flap is our preferred surgical approach in patients with anterior and large defects. On the other hand, in patients with lateral or posterior defect and poor medical condition, our preferred surgical approach is to either utilize the reconstruction plate alone or use it in conjunction with free tissue transfer to obtain an acceptable result without major complications.

 Finally, in terms of dental rehabilitation associated with mandibular reconstruction, it is important to remember the significance of tongue function in mastication; surgeons may consider using free flaps to replace missing portion of the anterior tongue if indicated. Also, the inclusion criteria for patient selection and the timing of dental implant placement should be carefully evaluated in order to avoid unforeseen prosthetic complications and diminish the chance of implant failure.

## Figures and Tables

**Table 1:** Characteristics of different osteocutaneous flaps.

Characteristics	Fibula	Scapula	Iliac crest	Radius
Reliability of skin paddle	+++ Perforator flap, thin but hairy	+++ Perforator flap, superior color match	+++ Perforator flap, too bulky	++++ Perforator flap, thin and flexible
Bone quality	+++ Bicortical bone, 20 cm	++ Unicortical bone, 14–17 cm	+++ Unicortical bone, 12 cm	++ Unicortical bone, 14 cm
Osseointegration of dental implants	+++	++	+++	+
Pedicle length	+++ 12–15 cm	++ 4–6 cm	++ 4–7 cm	++++ 20 cm
Sensory innervation	++++ Lateral sural cutaneous nerve	+ None	++ Lateral branch of the last thoracic nerve	++++ Anterior antebrachial cutaneous nerve
Donor site complication	++ Ankle instability, stiffness	++	++ Hernia, pain	++ Radial fracture
Two teams work	Yes	No	Yes	No

Best: ++++, worst: +.

**Table 2:** Outcomes of free osteocutaneous flap reconstruction.

First author	Year	Flap number	Success rate (%)	Bone union rate (%)	Osseointegrated dental implants number (%)	Diet (%)				Speech (%)				Aesthetic outcome (%)			
						Normal	Soft	Liquid	NG feeding	Normal	Near normal	Intelligible	Unintelligible	Excellent	Good	Fair	Poor
Foster	1999	Ilium 49	96	88	—	—	—	—	—	—	—	—	—	—	—	—	—
Cordeiro	1999	150	100	97	20 (7.5)	45	45	5	5	36	27	28	9	32	27	27	14
Shptizer	1999	Ilium 31 Fibula 48	100	—	—	32	61		6	84		13	3	61		32	6
						63	27		10	90		10	0	58		38	4
Shptizer	2000	Fibula 14	100	—	—	50	35.7		14.3	92.9		7.1	0	78.6		14.3	7.1
Hidalgo	2002	20	100	—	5 (25)	70	30	—	—	85		15		55	20	15	10
Vayada	2006	11	100	—	—	100	—	—	—	100		—	—	82		18	—
Virgin	2010	Fibula 117 Radius 57	96.6	—	1 (1)	22.6	50		27.4	—	—	—	—	—	—	—	—
			96.1		3 (5.8)	25.6	53.5		20.9								

## References

[1] Taylor GI, Watson N (1978). One-stage repair of compound leg defects with free, revascularized flaps of groin skin and iliac bone. *Plastic and Reconstructive Surgery*.

[2] Taylor GI, Townsend P, Corlett R (1979). Superiority of the deep circumflex iliac vessels as the supply for free groin flaps. Experimental work. *Plastic and Reconstructive Surgery*.

[3] Koshima I, Hosoda M, Ohta S (1994). Free vascularized iliac osteomusculocutaneous flaps based on the lateral circumflex femoral system for repair of large mandibular defects. *Annals of Plastic Surgery*.

[4] Taylor GI, Miller GDH, Ham FJ (1975). The free vascularized bone graft. A clinical extension of microvascular techniques. *Plastic and Reconstructive Surgery*.

[5] Hidalgo DA (1989). Fibula free flap: a new method of mandible reconstruction. *Plastic and Reconstructive Surgery*.

[6] Zlotolow IM, Huryn JM, Piro JD, Lenchewski E, Hidalgo DA (1992). Osseointegrated implants and functional prosthetic rehabilitation in microvascular fibula free flap reconstructed mandibles. *The American Journal of Surgery*.

[7] Wei FC, Seah CS, Tsai YC, Liu SJ, Tsai MS (1994). Fibula osteoseptocutaneous flap for reconstruction of composite mandibular defects. *Plastic and Reconstructive Surgery*.

[8] Wei FC, Chuang SS, Yim KK (1994). The sensate fibula osteoseptocutaneous flap: a preliminary report. *British Journal of Plastic Surgery*.

[9] O’Leary MJ, Martin PJ, Hayden RE (1994). The neurocutaneous free fibula flap in mandibular reconstruction. *Otolaryngologic Clinics of North America*.

[10] Kuo YR, Shih HS, Chen CC (2010). Free fibula osteocutaneous flap with soleus muscle as a chimeric flap for reconstructing mandibular segmental defect after oral cancer ablation. *Annals of Plastic Surgery*.

[11] Yang G-F, Chen P-J, Gao Y-Z (1997). Forearm free skin flap transplantation: a report of 56 cases. 1981. *British Journal of Plastic Surgery*.

[12] Soutar DS, Scheker LR, Tanner NSB, McGregor IA (1983). The radial forearm flap: a versatile method for intra-oral reconstruction. *British Journal of Plastic Surgery*.

[13] Swartz WM, Banis Newton JCED (1986). The osteocutaneous scapular flap for mandibular and maxillary reconstruction. *Plastic and Reconstructive Surgery*.

[14] Klotch DW, Prein J (1987). Mandibular reconstruction using AO plates. *American Journal of Surgery*.

[15] Kim PD, Blackwell KE (2007). Latissimus-serratus-rib free flap for oromandibular and maxillary reconstruction. *Archives of Otolaryngology - Head and Neck Surgery*.

[16] Cordeiro PG, Hidalgo DA (1995). Conceptual considerations in mandibular reconstruction. *Clinics in Plastic Surgery*.

[17] Anthony JP, Rawnsley JD, Benhaim P, Ritter EF, Sadowsky SH, Singer MI (1995). Donor leg morbidity and function after fibula free flap mandible reconstruction. *Plastic and Reconstructive Surgery*.

[18] Schusterman MA, Reece GP, Kroll SS, Weldon ME (1991). Use of the AO plate for immediate mandibular reconstruction in cancer patients. *Plastic and Reconstructive Surgery*.

[19] Boyd JB, Mulholland RS, Davidson J (1995). The free flap and plate in oromandibular reconstruction: long-term review and indications. *Plastic and Reconstructive Surgery*.

[20] Shpitzer T, Gullane PJ, Neligan PC (2000). The free vascularized flap and the flap plate options: comparative results of reconstruction of lateral mandibular defects. *Laryngoscope*.

[21] Boyd JB (1994). Use of reconstruction plates in conjunction with soft-tissue free flaps for oromandibular reconstruction. *Clinics in Plastic Surgery*.

[22] Hanasono MM, Zevallos JP, Skoracki RJ, Yu P (2010). A prospective analysis of bony versus soft-tissue reconstruction for posterior mandibular defects. *Plastic and Reconstructive Surgery*.

[23] Foster RD, Anthony JP, Sharma A, Pogrel MA (1999). Vascularized bone flaps versus nonvascularized bone grafts for mandibular reconstruction: an outcome analysis of primary bony union and endosseous implant success. *Head and Neck*.

[24] Cordeiro PG, Disa JJ, Hidalgo DA, Hu QY (1999). Reconstruction of the mandible with osseous free flaps: a 10-year experience with 150 consecutive patients. *Plastic and Reconstructive Surgery*.

[25] Shpitzer T, Neligan PC, Gullane PJ (1999). The free iliac crest and fibula flaps in vascularized oromandibular reconstruction: comparison and long-term evaluation. *Head and Neck*.

[26] Hidalgo DA, Pusic AL (2002). Free-flap mandibular reconstruction: a 10-year follow-up study. *Plastic and Reconstructive Surgery*.

[27] Vayvada H, Mola F, Menderes A, Yilmaz M (2006). Surgical management of ameloblastoma in the mandible: segmental mandibulectomy and immediate reconstruction with free fibula or deep circumflex iliac artery flap (evaluation of the long-term esthetic and functional results). *Journal of Oral and Maxillofacial Surgery*.

[28] Virgin FW, Iseli TA, Iseli CE (2010). Functional outcomes of fibula and osteocutaneous forearm free flap reconstruction for segmental mandibular defects. *Laryngoscope*.

[29] Farhadi J, Valderrabano V, Kunz C, Kern R, Hinterman B, Pierer G (2007). Free fibula donor-site morbidity: clinical and biomechanical analysis. *Annals of Plastic Surgery*.

[30] Zimmermann CE, Börner BI, Hasse A, Sieg P (2001). Donor site morbidity after microvascular fibula transfer. *Clinical oral investigations*.

[31] Hsieh CH, Cheung SM, Sun CK (2010). Evaluation of the ankle function following reconstruction of the donor defect with a split fibular bone after a vascularized fibular flap transfer. *Archives of Orthopaedic and Trauma Surgery*.

[32] Babovic S, Johnson CH, Finical SJ (2000). Free fibula donor-site morbidity: the Mayo experience with 100 consecutive harvests. *Journal of Reconstructive Microsurgery*.

[33] Chen Y-C, Valerio IL, Chien C-Y, Jeng S-F Pedicled mandible myo-osseous flaps combined with free skin flaps for reconstruction of complex lateral mandibular defects.

[34] Jeng SF, Kuo YR, Wei FC, Su CY, Chien CY (2005). Reconstruction of extensive composite mandibular defects with large lip involvement by using double free flaps and fascia lata grafts for oral sphincters. *Plastic and Reconstructive Surgery*.

[35] Lin PY, Kuo YR, Chien CY, Jeng SF (2009). Reconstruction of head and neck cancer with double flaps: comparison of single and double recipient vessels. *Journal of Reconstructive Microsurgery*.

[36] Pruyn JFA, De Jong PC, Bosman LJ (1986). Psychosocial aspects of head and neck cancer—a review of the literature. *Clinical Otolaryngology and Allied Sciences*.

[37] Roumanas ED, Garrett N, Blackwell KE (2006). Masticatory and swallowing threshold performances with conventional and implant-supported prostheses after mandibular fibula free-flap reconstruction. *Journal of Prosthetic Dentistry*.

[38] Curtis DA, Plesh O, Miller AJ (1997). A comparison of masticatory function in patients with or without reconstruction of the mandible. *Head and Neck*.

[39] Beumer J, Roumanas E, Nishimura R (1995). Advances in osseointegrated implants for dental and facial rehabilitation following major head and neck surgery. *Seminars in Surgical Oncology*.

[40] Raoul G, Ruhin B, Briki S (2009). Microsurgical reconstruction of the jaw with fibular grafts and implants. *The Journal of craniofacial surgery*.

[41] Ghana JS, Chang Y-M, Wei F-C (2004). Segmental mandibulectomy and immediate free fibula osteoseptocutaneous flap reconstruction with endosteal implants: an ideal treatment method for mandibular ameloblastoma. *Plastic and Reconstructive Surgery*.

